# Type 1 Diabetes Risk Variants Reduce Beta Cell Function

**DOI:** 10.3390/genes16020172

**Published:** 2025-01-29

**Authors:** Wiktoria Ratajczak, Angus G. Jones, Sarah D. Atkinson, Catriona Kelly

**Affiliations:** 1 Personalised Medicine Centre, School of Medicine, Ulster University, C-TRIC Building, Altnagelvin Hospital Campus, Glenshane Road, Derry BT48 7JL, UK; ratajczak-w@ulster.ac.uk; 2 University of Exeter Medical School, RILD Building, RD&E Hospital Wonford, Barrack Road, Exeter EX1 2LU, UK; angus.jones@exeter.ac.uk; 3 School of Biomedical Sciences, Ulster University, Cromore Road, Coleraine BT52 1SA, UK; sa.atkinson@ulster.ac.uk

**Keywords:** type 1 diabetes, risk variants, β cells, inflammation

## Abstract

Introduction: The variants rs10517086 and rs1534422 are predictive of type 1 diabetes mellitus (T1DM) development and poor residual β cell function within the first year of diagnosis. However, the mechanism by which risk is conferred is unknown. We explored the impact of both variants on β cell function in vitro and assessed their relationship with C-peptide in people with T1DM and type 2 diabetes mellitus (T2DM). Methods: Using CRISPR/Cas9, the variants were introduced into a β cell line (BRIN-BD11) and a T cell line (Jurkat cells) from which the conditioned media was applied to otherwise healthy β cells to model the inflammatory environment associated with these variants. Results: Both variants significantly reduced glucose-stimulated insulin secretion, increased production of pro-inflammatory cytokines and reduced expression of several β cell markers and transcription factors (*KCNJ11*, *KCNQ1*, *SCL2A2*, *GCK*, *NKX6.1*, *Pdx1 NGN3*). However, *HNF1A* was significantly upregulated in the presence of both variants. We subsequently silenced *HNF1A* in variant expressing BRIN-BD11 cells using siRNA and found that gene expression profiles were normalised. Induction of each variant significantly increased expression of the lncRNAs they encode, which was normalised upon *HNF1A* silencing. Analysis of the DARE (Diabetes Alliance for Research in England) study revealed an association of rs10517086_A genotype with C-peptide in 153 individuals with T1DM, but not in 417 people with T2DM. Conclusions: These data suggest that rs1534422 and rs10517086 exert multiple insults on the β cell through excessive upregulation of *HNF1A* and induction of pro-inflammatory cytokines, and highlight their utility as prognostic markers of β cell function.

## 1. Introduction

Type 1 diabetes mellitus (T1DM) is a multifactorial autoimmune disease characterised by a gradual loss of insulin-secreting pancreatic β cells as a function of non-mutually exclusive events, including destruction by T-cell mediated responses and de-differentiation [1]. There are several mechanisms that lead to β cell mass decrease, such as de-differentiation and trans-differentiation; however, the characteristic destruction of β cells in T1D occurs largely by apoptosis [2,3]. Prior to onset of T1D, the Islets of Langerhans are subjected to an autoimmune assault by an inflammatory infiltrate consisting of autoreactive T lymphocytes, macrophages, B lymphocytes and dendritic cells [1]. Factors such as low thymic expression of islet antigens, poor binding of non-transcriptionally modified islet autoantigens to MHC classes I and II and T cell intrinsic resistance to apoptosis have been named as reasons for bypassing the thymic control [1]. Invading immune cells produce a large amount of the pro-inflammatory cytokines IL-1β, TNF-α, and IFN-γ, which induce β cell apoptosis through activation of the transcription factors NF-κB and STAT-1 [4]. Activation of the NF-κB pathway leads to formation of nitric oxide, further chemokine production and reduction in endoplasmic reticulum (ER) calcium stores, which lead to downstream apoptosis [2]. Exposure to continuous high glucose concentrations leads to glucose hypersensitisation and apoptosis via NF-κB–independent mechanisms [2]. By the time diagnosis is made, there is a 70–80% reduction in β cell mass, which translates to reductions in insulin production [5].

The causes of T1DM are not fully understood, despite the identification of several risk factors [6]. The probability of developing T1DM is increased by human leukocyte antigens (HLA)-DQA1, HLA-DQB1 and HLA-DRB1 gene variants [7]. Although variants in the HLA genes account for the majority of T1DM risk, GWAS studies have identified several other variants that confer significant risk for disease development [7]. The variants rs10517086 (long intergenic non-protein coding RNA 2357 (*LINC02357*) and rs1534422 (MIR3681 host gene (*MIR3681HG*)) were linked with an increased risk of progression to T1DM in the TEDDY (The Environmental Determinants of Diabetes in the Young) study [8,9]. It was demonstrated that the rs10517086 variant displays stage- and age-related heterogeneity, with increased risk of T1DM and islet autoimmunity (IA) development in children under the age of 2 years [10]. Several *in silico* studies have made a connection between the rs1534422 variant, IA and T1D, as well as other autoimmune diseases, such as multiple sclerosis [8,11,12].

Despite identification of rs10517086 and rs1534422 as risk variants for progression to T1DM and early indications that they may offer prognostic value for longer-term β cell function, little is known about the mechanisms by which risk is conferred [13,14]. It is currently unclear whether these variants directly impact β cell function and/or survival, or whether they create an inflammatory environment that is ultimately detrimental to the β cell. Using gene editing approaches, we sought to determine the functional impact of these variants on β cell models in vitro.

## 2. Methods

We assessed the functional impact of rs10517086 and rs1534422 in a β cell line using CRISPR/Cas9. We then assessed the relationship between these variants and residual c-peptide in 153 patients with T1D and 417 with T2D.

### 2.1. Cell Culture and Treatment

All experiments and optimisation protocols were performed in an insulin-secreting BRIN-BD11 cell line, which was purchased from the European Collection of Authenticated Cell Cultures (ECACC; Salisbury, UK) and a Jurkat T-cell line, also acquired from ECACC. All cells were cultured and maintained according to the suppliers’ directions. Functional studies were performed within the passage number range recommended by the manufacturer. BRIN-BD11 cells were routinely passaged until passage 35, and Jurkat T cells until passage 20.

### 2.2. CRISPR/Cas9 Construct Generation and Transfection

Guidance RNA (gRNA) and ssOligo donor (5′-3′) for rs1534422 and rs10517086 were designed, manufactured and purchased from Thermo Fisher Scientific (Loughborough, UK). A transfection ready donor strand was generated using TrueTag DNA GFP Donor Kit (Thermo Fisher Scientific) according to the manufacturer’s recommendations (Table 1).

A total of 5 × 10^4^ BRIN-BD11 cells were seeded per well of a 24-well plate and allowed to attach overnight, prior to transfection experiments. Jurkat T cells were seeded at a density of 5 × 10^4^ per well of a 24-well plate and CRISPR transfection was performed immediately. A total of 1250 ng TrueCut Cas 9 Protein v2 (Thermo Fisher Scientific) 280 ng gRNA, 600 ng donor strand and 1.5 μL lipofectamine Cas9 reagent (Thermo Fisher Scientific) were added to a tube containing 25 μL optiMem I reduced media (Gibco, Loughborough, UK) and 2.5 μL Lipofectamine™ CRISPRMAX (Thermo Fisher Scientific). Tubes were allowed to incubate for 15 min at room temperature to allow the formation of transfection complexes, and thereafter, added dropwise to the cells. BRIN-BD11 and Jurkat T cells were incubated in a humidified 37 °C, 5% CO_2_ incubator for 72 h. After 72 h, the gene editing efficiency was verified, and cells expanded for downstream applications.

### 2.3. Verification of CRISPR/Cas9 Efficiency

The success of CRISPR/Cas9 transfections was confirmed by three methods (Figure S1, Electronic Supplementary Material (ESM)). The presence of a fluorescent GFP tag was visually confirmed with a fluorescent microscope. Part of the cell population was maintained for functional studies and the remainder was harvested. DNA was extracted using the Qiagen QIAMP DNA mini kit (Manchester, UK) and the presence of variants confirmed through end point genotyping with independent TaqMan genotyping probes (rs1534422, assay ID: C____418159_10; rs10517086, assay ID: C__30562980_20; Thermo Fisher Scientific) according to the manufacturer’s recommendations. The restriction digest enzymes TspRI (rs1534422) and HPY188III (rs10517086), obtained from NEB (Hitchin, UK), were used according to the manufacturer’s instructions to investigate the presence or absence of the wild-type sequence. The analysis was carried out via gel electrophoresis on a 2% agarose gel.

### 2.4. Cell Treatments

BRIN-BD11 cells expressing rs1534422 or rs10517086 were tested in the presence or absence of 10 ng/mL TNF-α for 1 h to induce an inflammatory response and differences were compared with negative (vehicle) controls. Wild-type BRIN-BD11 cells were also treated with conditioned media from variant expressing Jurkat T cells, which were activated and matured through exposure to 50 ng/mL PMA for 24 h prior to the collection of cell-free conditioned media.

### 2.5. Assessment of Viability, Cytotoxicity and Apoptosis

The Apotox-Glo™ Triplex Assay by Promega (Southampton, UK) allows for concurrent measurements of cell viability, cytotoxicity and apoptosis. Fluorogenic peptide substrate (bis-AAF-R110 Substrate) was used to measure dead cell protease activity, to assess viability and cytotoxicity of treated cells. Caspase-3/7 and Ultra-Glo™ Recombinant Thermostable Luciferase luminesce measurements were used as a direct measure of apoptosis. All experiments were performed as previously described [15].

### 2.6. Measurement of Glucose-Induced Insulin Secretion

BRIN-BD11 cells were exposed to basal (1.1 mM), and stimulatory (16.7 mM) concentrations of D-Glucose for 40 min, as previously described [15]. Insulin release was measured using a Mercodia (Uppsala, Sweden) Ultrasensitive Rat insulin ELISA kit, according to the manufacturer’s instructions.

### 2.7. Quantitative Real Time PCR (qPCR)

Total RNA was extracted, quantified, and converted to cDNA, as previously described [11]. qPCR was performed on a Lightcycler 480 System (Roche, Welwyn Garden City, UK) using custom designed probes (See Table 2) and master mix, as previously described [11]. Due to difficulties in obtaining custom probes for two lncRNAs, oligo primers were designed using Primer Blast, https://www.ncbi.nlm.nih.gov/tools/primer-blast/ (accessed 20 October 2024; Table 3). qPCR was performed using 20 ng cDNA for each sample with SYBR Green Master Mix (Roche) in a 20 μL reaction with the following parameters: [50 °C × 2 min, 95 °C × 10 min, 40 (95 °C × 15 s, 60 °C × 1 min), 95 °C × 15 s, 60 °C × 15 s, 95 °C × 15 s]. Relative mRNA expression was determined using the 2^ΔCt^ method.

### 2.8. Determination of Cytokine Concentration

Following successful completion of the CRISPR transfection process, fresh culture medium was added to Jurkat T cells and BRIN-BD11 cells and collected 24 h later. A V-plex proinflammatory panel 2 kit (Mesoscale, MD, USA) was used to measure the levels of nine cytokines that are important in inflammatory responses and immune system regulation, according to the manufacturer’s recommendations.

### 2.9. Silencing of HNF1A

Wild-type and CRISPR-edited BRIN-BD11 were transfected using siRNA against *HNF1A* (Silencer Select siRNA; Thermofisher, Loughborough, UK). CRISPR-edited cells were first maintained in fresh media for 48 h, following successful variant insertion (See Figure S2, ESM). Then 100 ng *HNF1A* siRNA and 2.5 μL lipofectamine 2000 (Qiagen, Manchester, UK) were added to 100 μL serum free RPMI media (Gibco, Loughborough, UK). A negative control consisted of 100 ng scrambled siRNA (AllStars Negative Control siRNA; Qiagen, Manchester, UK), 2.5 μL lipofectamine, and 100 μL serum free RPMI media. Tubes were allowed to incubate for 10 min at room temperature and added dropwise to the cells. BRIN-BD11 cells were maintained in the presence of transfection complexes for 72 h without media change. Knockdown was confirmed at the mRNA level by qPCR (Figure S3, ESM).

### 2.10. Analysis of Participant Data

Data from the Diabetes Alliance for Research in England (DARE) cohort was analysed to assess the prevalence of variants in those with a confirmed diagnosis of T1D or T2D and the association of genotype with C-peptide. DARE recruited peopled aged >=18 living with diabetes in the South West of England. All participants recruited to the DARE cohort had a clinical diagnosis of T1D or T2D, and available C-peptide and genotype data were analysed in this study. C-peptide was measured on non-fasting (random) samples, as previously described [16]. Where multiple samples were available (70% of participants, median 3 values per participant), the median value was used.

The DARE study was approved by the South West Ethics Committee (UK). Participants gave informed consent. The datasets analysed in this study were accessed through an application to the Peninsula Research Bank, which is managed by the NIHR Exeter Clinical Research Facility.

### 2.11. Data Analysis

Statistical analysis on data was performed using GraphPad PRISM (La Jolla, CA, USA; version 7). Data from cell culture experiments are presented as mean ± SEM for a given number of observations (n), as indicated in the Figures. Following assessment of normality, differences between cells expressing either variant and their corresponding controls were compared using the 2-tailed Student’s *t*-test. C-peptide data are presented as box plots with min and max whiskers. We compared the relationship between C-peptide and genotype using Mann–Whitney tests. Significance was accepted at *p* < 0.05 for all analysis.

## 3. Results

### 3.1. rs1534422 Increases β Cell Apoptosis

**Figure 1:**
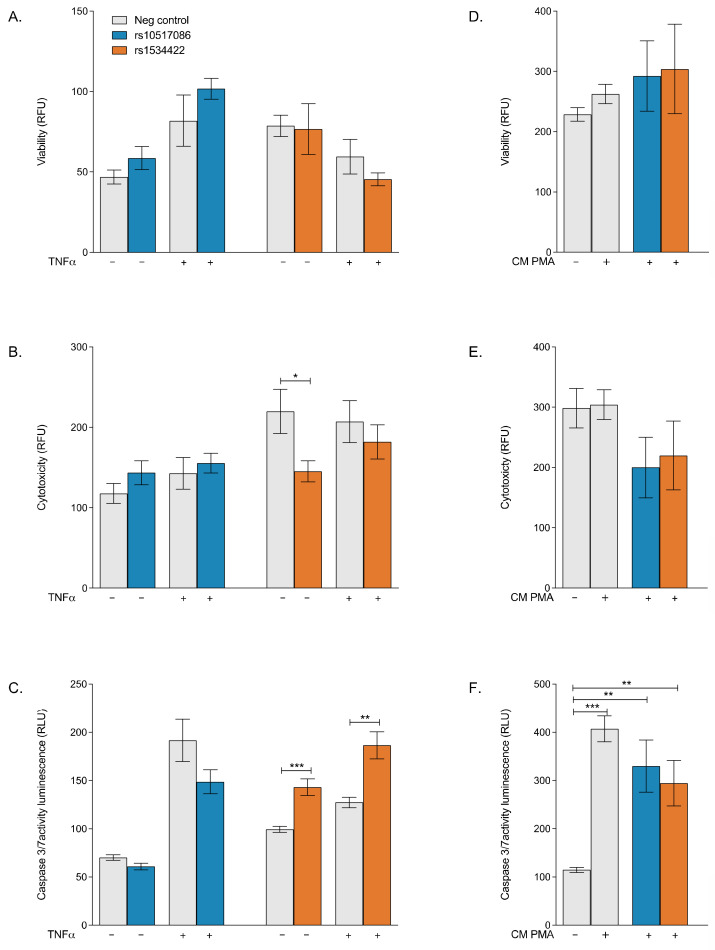
Impact of T1D risk variants on viability, cytotoxicity and apoptosis. rs10517086 and rs1534422 were induced in both BRIN-BD11 cells and Jurkat cells. BRIN-BD11 cells were exposed to 10 ng/mL TNFa for 1 h (**A**–**C**) or conditioned media from Jurkat cells exposed to 10 nM PMA (CM PMA) for 24 h (**D**–**F**). The impact on cell viability (**A**,**D**), cytotoxicity (**B**,**E**) and apoptosis (**C**,**F**) was assessed using the ApoTox-Glo Triplex Assay (Promega). Data are presented as mean ± SEM (n = 5–6 for all experiments). * *p* < 0.05, ** *p* < 0.01, *** *p* < 0.001 compared with corresponding control (unpaired *t*-test). TNFa, tumour necrosis factor a; PMA, phorbol 12-myristate 13-acetate.

Cell viability, cytotoxicity and apoptosis were assessed using the AptoTox Glo™ assay. The presence of rs10517086 did not significantly impact cell viability (Figure 1A), cytotoxicity (Figure 1B) or apoptosis (Figure 1C) in BRIN-BD11 cells, regardless of whether cells were treated with TNF-α or not. rs1534422 did not affect cell viability (Figure 1A) but did result in a modest reduction in cytotoxicity in the absence of TNF-α treatment (34 ± 0.13% reduction, *p* < 0.05, Figure 1B), a trend that was not significant in the presence of TNF-α stimulation (12.26 ± 0.21% reduction, ns, Figure 1B). The presence of rs1534422 caused significant increases in apoptosis in BRIN-BD11 cells, irrespective of TNF-α treatment (1.44 ± 0.09%-fold increase in absence of TNF-α, *p* < 0.001, Figure 1C; 1.47 ± 0.14%-fold increase in presence of TNF-α, *p* < 0.01, Figure 1C). The addition of conditioned media from variant expressing Jurkat cells had no impact on cell viability, cytotoxicity or apoptosis beyond the level of the PMA control (Figure 1E,F).

### 3.2. rs10517086 and rs1534422 Reduce Glucose-Stimulated Insulin Secretion

**Figure 2:**
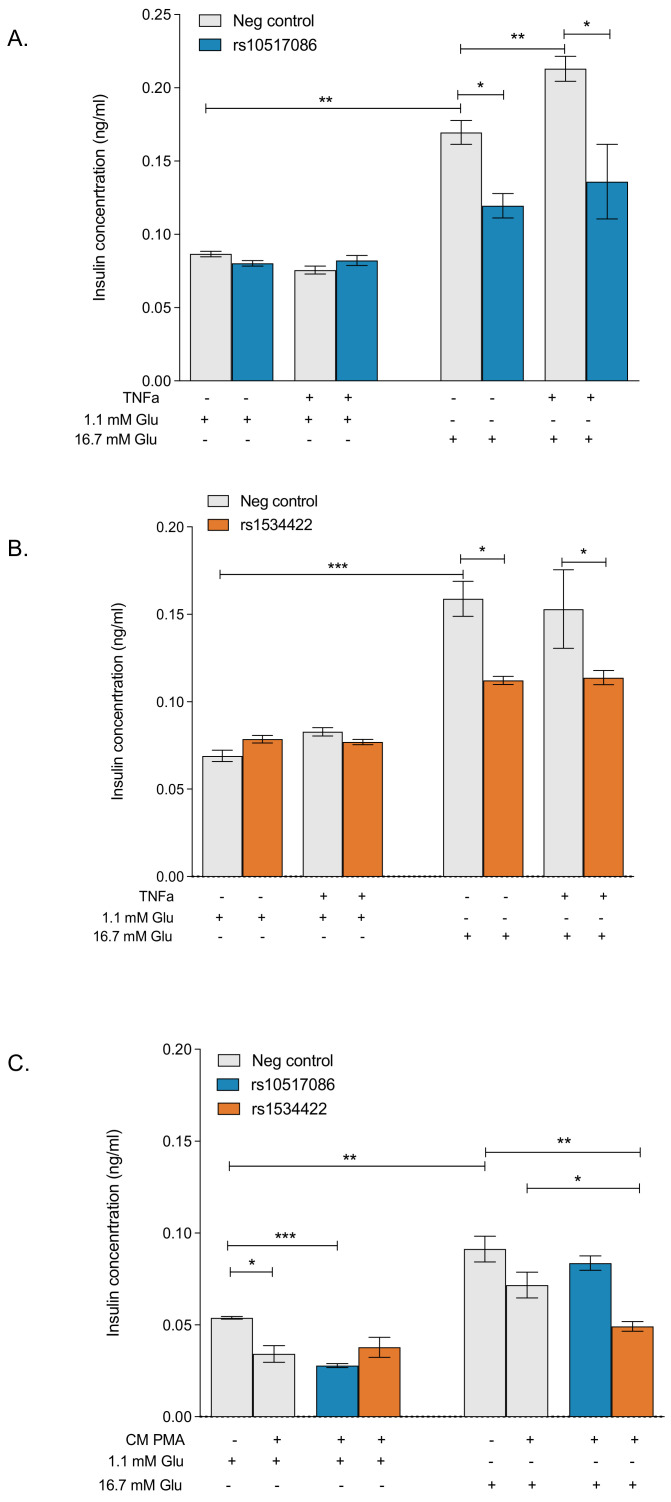
Effect of T1D risk variants on glucose-stimulated insulin secretion. rs10517086 and rs1534422 were induced in both BRIN-BD11 cells and Jurkat cells. BRIN-BD11 cells were exposed to 10 ng/mL TNFa for 1 h (**A**,**B**) or conditioned media from Jurkat cells exposed to 10 nM PMA (CM PMA) for 24 h (**C**). The impact on basal (1.1 mM) and stimulated (16.7 mM) glucose induced insulin secretion was determined by ELISA. Data are presented as mean ± SEM (n = 4–6 for all experiments). * *p* < 0.05, ** *p* < 0.01, *** *p* < 0.001 compared with corresponding control (unpaired *t*-test). TNFa, tumour necrosis factor a; PMA, phorbol 12-myristate 13-acetate.

**Figure 3:**
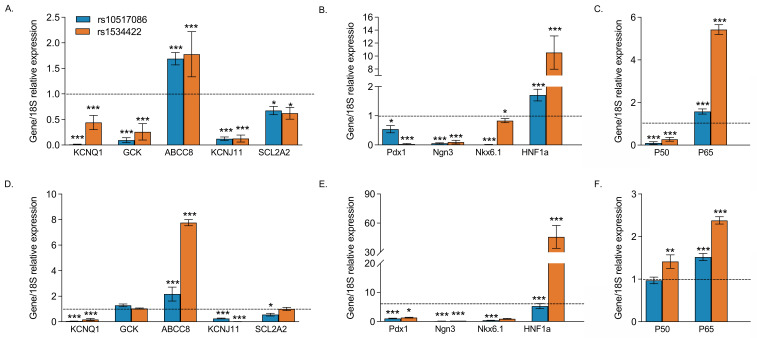
Impact of T1D risk variants on expression of islet and inflammatory genes. rs10517086 and rs1534422 were induced in both BRIN-BD11 cells and Jurkat cells. BRIN-BD11 cells were exposed to 10 ng/mL TNFa for 1 h (**A**–**C**) or conditioned media from Jurkat cells exposed to 10 nM PMA (CM PMA) for 24 h (**D**–**F**). The impact on the mRNA expression of β cell markers (**A**,**D**), islet transcription factors (**B**,**E**) and inflammatory markers (**C**,**F**) was assessed by qPCR and standardised against the corresponding control using 2^ΔCt^. Hashed lines present expression levels in control samples. Data are presented as mean ± SEM (n = 3–4 for all experiments). * *p* < 0.05, ** *p* < 0.01, *** *p* < 0.001 compared with corresponding control (unpaired *t*-test).

The effects of rs10517086 and rs1534422 on insulin secretion in response to basal (1.1 mM) and stimulatory (16.7 mM) glucose concentrations were measured by ELISA and compared with negative (vehicle) controls. BRIN-BD11 cells were exposed to 10 ng/mL TNF-α for 1 h prior to acute exposure to glucose. Neither variant affected basal insulin secretion, regardless of TNF-α treatment (Figure 2A,B). However, both variants resulted in significant reductions (*p* < 0.05) in insulin release in response to stimulatory concentrations of glucose. This was again irrespective of TNF-α treatment (Figure 2A,B). Knock-in of rs10517086 resulted in a 29.5 ± 8% decrease in the absence of TNF-α (*p* < 0.05) and a 36.2 ± 3% decrease in the presence of TNF-α (*p* < 0.05, Figure 3A). Similarly, expression of rs1534422 caused a 29.3 ± 2% decrease in the absence of TNF-α (*p* < 0.05) and a 25.6 ± 4% decrease in the presence of TNF-α (*p* < 0.05, Figure 3B).

The impact of conditioned media from variant-containing Jurkat cells on insulin release from wild-type BRIN-BD11 cells was varied (Figure 2C). Conditioned media from PMA activated wild-type Jurkat cells was sufficient to trigger reductions in insulin release in response to basal glucose concentrations (*p* < 0.05, Figure 2C) and this was not further exacerbated when variants were introduced to the cells (Figure 2C). rs10517086 had no effect on insulin secretion in response to stimulatory concentrations of glucose (Figure 2C); however, rs1534422 was associated with a significant reduction in insulin secretion in this instance (31.4 ± 3% reduction, *p* < 0.05, Figure 3C).

### 3.3. rs10517086 and rs1534422 Alter β Cell Transcriptional Profiles

The mRNA expression of β cell markers (*KCNQ1*, *GCK*, *ABCC8*, *KCNJ11*, *SCL2A2*), transcription factors regulating β cell maturation and function (*HNF1A*, *PDX1*, *NKX6.1 NGN3*) and inflammatory markers (NF-κB subunits, *NKFB1* and *RelA*) were examined in variant expressing BRIN-BD11 cells or wild-type BRIN-BD11 cells exposed to conditioned media from variant-containing Jurkat cells (Figure 3). The majority of β cell markers and transcription factors were down-regulated (*p* < 0.05–0.001), with two notable exceptions: both *HNF1A* and *ABCC8* were significantly upregulated (*p <* 0.001). Additionally, the NF-κB subunit *RelA* was significantly upregulated in the presence of either variant (*p* < 0.001). These findings were consistent, regardless of whether variants were expressed directly in BRIN-BD11 cells (Figure 3A–C) or whether wild-type BRIN-BD11 cells were exposed to conditioned media from variant expressing Jurkat cells (Figure 3D–F).

### 3.4. rs10517086 and rs1534422 Alter the Concentration of Secreted Inflammatory Cytokines

**Figure 4:**
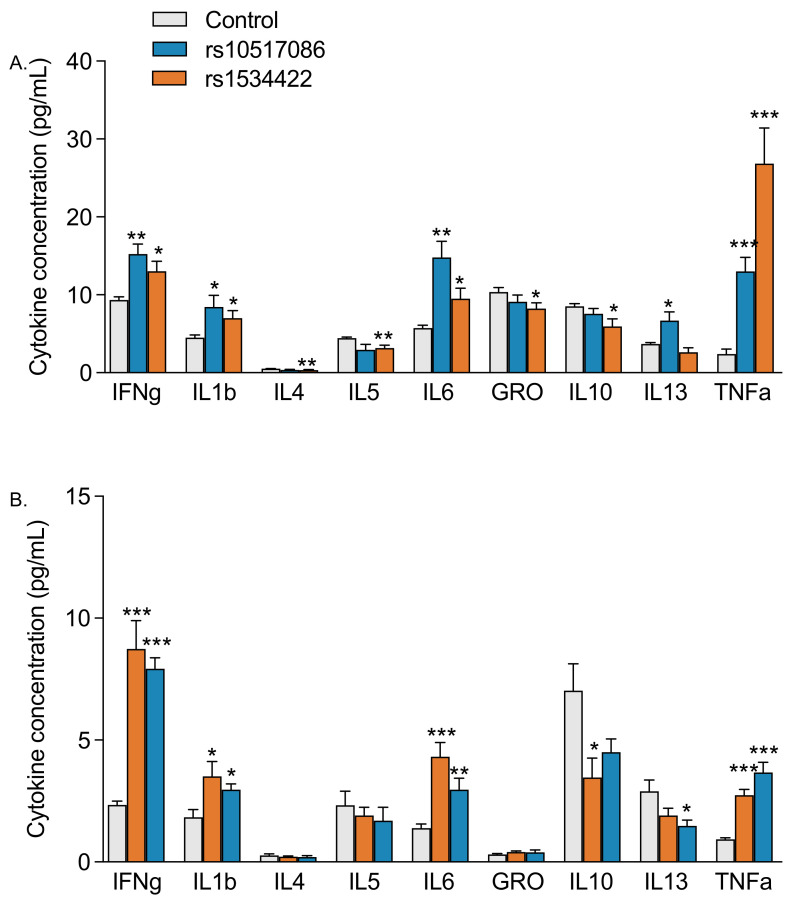
Impact of T1D risk variants on the secretion of inflammatory markers. rs10517086 and rs1534422 were induced in both BRIN-BD11 cells and Jurkat cells. BRIN-BD11 cells were exposed to 10 ng/mL TNFa for 1 h (**A**) and Jurkat cells exposed to 10 nM PMA (CM PMA) for 24 h (**B**) and the media collected. The impact of variants on the profile of secreted cytokines was determined by the Mesoscale V-PLEX Proinflammatory Panel. Data are presented as mean ± SEM (n = 6 for all experiments). * *p* < 0.05, ** *p* < 0.01, *** *p* < 0.001 compared with corresponding control (unpaired *t*-test). TNFa, tumour necrosis factor a; PMA, phorbol 12-myristate 13-acetate.

The Mesoscale V-PLEX Proinflammatory Panel was used to determine the concentration of cytokines (IFN-γ, IL-1β, IL-4, IL-5, IL-6, IL-10, IL-13, TNF-α, KC/GRO) in media obtained from variant expressing BRIN-BD11 cells (Figure 4A) and Jurkat T cells (Figure 4B). The presence of rs10517086 and rs1534422 variants had a moderate but significant down-regulatory effect on the secretion of all investigated anti-inflammatory cytokines and a significant upregulatory effect on the secretion of pro-inflammatory cytokines (*p* < 0.05–0.001, Figure 4A,B). These changes in the secretory patterns were consistent, regardless of whether variants were expressed in BRIN-BD11 cells or Jurkat T cells.

### 3.5. Silencing of HNF1A in rs10517086 and rs1534422 Expressing BRIN-BD11 Cells Alleviates Apoptosis

**Figure 5:**
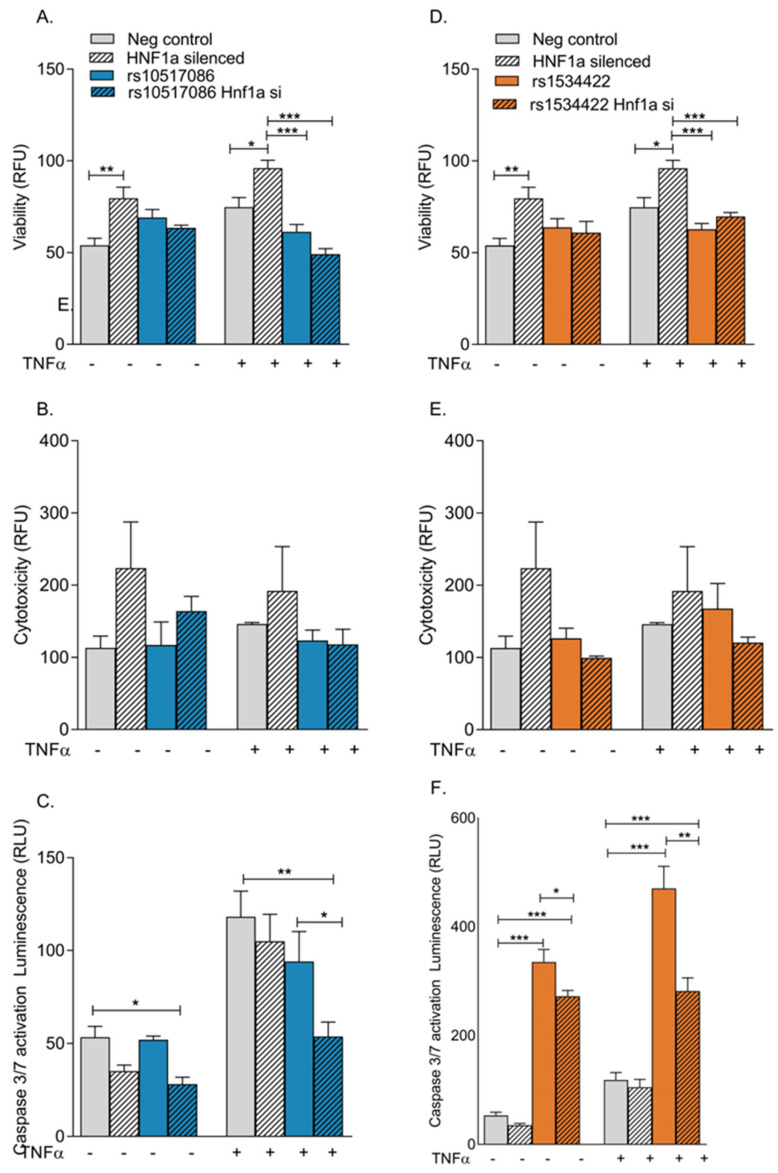
The impact of *HNF1A* silencing on viability, cytotoxicity and apoptosis of cells expressing T1D risk variants. rs10517086 and rs1534422 were induced in BRIN-BD11 cells, the cells were then treated with 100 ng siRNA against *HNF1A* or a negative control (scrambled siRNA) and then exposed to 10 ng/mL TNFa for 1 h. The impacts on cell viability (**A**,**D**), cytotoxicity (**B**,**E**) and apoptosis (**C**,**F**) were assessed using the ApoTox-Glo Triplex Assay (Promega). Data are presented as mean ± SEM (n = 4–6 for all experiments). * *p* < 0.05, ** *p* < 0.01, *** *p* < 0.001 compared with corresponding control (unpaired *t*-test). TNFa, tumour necrosis factor a.

*HNF1A* was silenced in wild-type BRIN-BD11, rs1534422 expressing BRIN-BD11 and rs10517086 expressing BRIN-BD11 cells. The consequences of *HNF1A* silencing on cell viability, cytotoxicity and apoptosis were confirmed using the AptoTox Glo™ assay. Following transfection, the cells were tested in the presence or absence of 10 ng/mL TNF-α for 1 h and differences compared with negative controls. Significant changes in cell viability and cytotoxicity were not observed, regardless of TNF-α stimulation in *HNF1A* silenced rs10517086 and rs1534422 expressing BRIN-BD11 cells (Figure 5A–D).

Silencing of *HNF1A* resulted in a significant decrease in apoptosis in rs10517086 expressing BRIN-BD11 cells in both the absence (*p* < 0.05) and presence (*p* < 0.01) of TNF-α (Figure 5C), when compared with the scrambled siRNA control. Silencing of *HNF1A* in rs1534422 expressing BRIN-BD11 cells led to a significant decrease in apoptosis in the absence (*p* < 0.05) and presence (*p* < 0.01) of TNF-α, when compared to rs1534422 expressing BRIN-BD11 cells alone (Figure 5F).

### 3.6. Silencing of HNF1A in rs10517086 and rs1534422 Expressing BRIN-BD11 Cells Normalises β Cell Gene Expression

**Figure 6:**
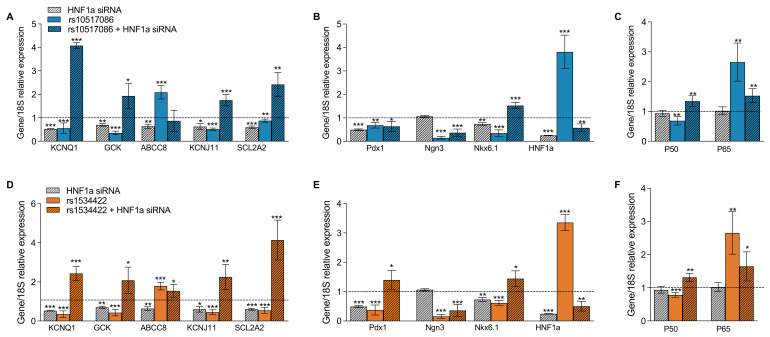
Impact of HNF1A silencing in cells expressing T1D risk variants on expression of islet and inflammatory genes. rs10517086 and rs1534422 were induced in BRIN-BD11 cells, which were subsequently treated with 100 ng siRNA against *HNF1A* or a negative control (scrambled siRNA) and then exposed to 10 ng/mL TNFa for 1 h. The impact on the mRNA expression of β cell markers (**A**,**D**), islet transcription factors (**B**,**E**) and inflammatory markers (**C**,**F**) was assessed by qPCR and standardised against the corresponding control using 2^ΔCt^. Hashed lines present expression levels in control samples. Data are presented as mean ± SEM (n = 4 for all experiments). * *p* < 0.05, ** *p* < 0.01, *** *p* < 0.001 compared with corresponding control (unpaired *t*-test).

The expressions of β cell markers, transcription factors and inflammatory markers were investigated in the rs1534422 and rs10517086 expressing BRIN-BD11 after *HNF1A* silencing (Figure 6). Silencing of *HNF1A* in wild-type BRIN-BD11 cells led to reductions in the expression of all investigated β cell markers (*p* < 0.05–0.001, Figure 6A) and almost all transcription factors (*p* < 0.05–0.001, Figure 6B), with the exception of *Ngn3*. The inflammatory markers *RelA* and *NKFB1* were not affected by *HNF1A* silencing alone (Figure 6C). Silencing of *HNF1A* in rs1534422 and rs10517086 expressing BRIN-BD11 cells led to normalisation of gene expression, when compared with cells expressing the variants alone (*p* < 0.05–0.001, Figure 6A–F).

### 3.7. rs10517086 and rs1534422 Increase Expression of Encoded lncRNAs

**Figure 7:**
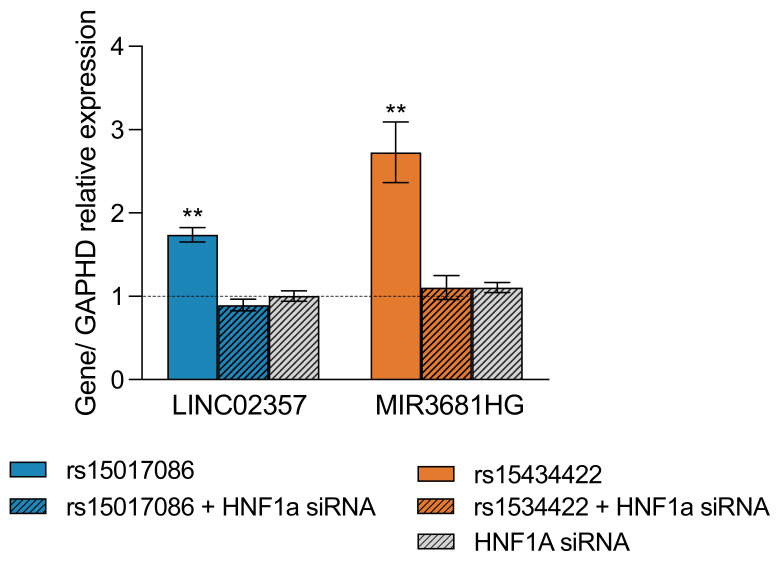
rs1534422 and increase expression of encoded lncRNAs. rs10517086 and rs1534422 were induced in BRIN-BD11 cells. The expression of *LINC023457* (encoded by rs10517086) and *MIR3681HG* (encoded by rs1534422) were investigated by qPCR in the presence or absence of 100 ng siRNA against *HNF1A* or a negative control (scrambled siRNA). Expression was standardized against the corresponding control (represented by the hashed line) using 2^ΔCt^. Data are presented as mean ± SEM (n = 3 for all experiments). ** *p* < 0.01, compared with the corresponding control (unpaired *t*-test).

The variant rs10517086 is located within *LINC023557,* and rs1534422 is in *MIR3681HG*. The expression of both genes was investigated in rs10517086 and rs1534422 expressing BRIN-BD11, before and after *HNF1A* silencing (Figure 7). Both lncRNAs were significantly upregulated upon induction of their corresponding variant (LINC023557: 1.79 ± 0.09-fold increase, *p* < 0.01; MIR3681HG: 2.73 ± 0.3-fold increase, *p* < 0.01). *HNF1A* silencing alone had no impact on the expression of either lncRNA; however, silencing of *HNF1A* in BRIN-BD11 cells expressing rs10517086 or rs1534422 resulted in normalisation of the expression of *LINC023557* and *MIR3681HG,* respectively.

### 3.8. Impact of T1D Risk Variants in Individuals from the DARE Study

**Figure 8:**
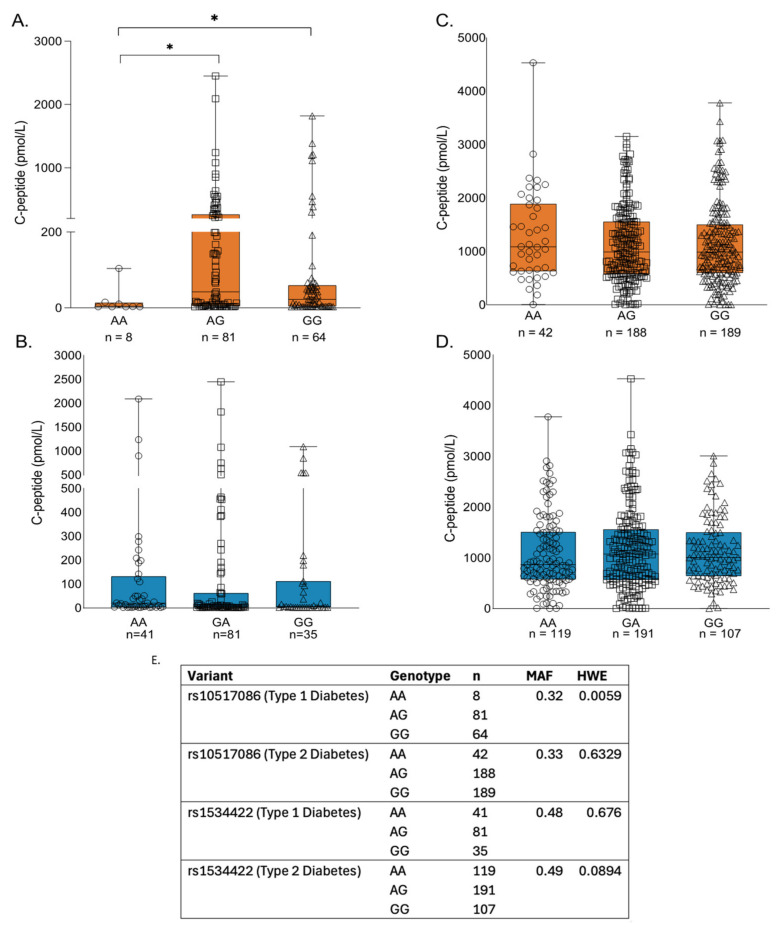
Relationship of T1D risk variants with C-peptide in individuals from the DARE study. The relationship with C-peptide of rs10517086 (**A**,**C**) and rs1534422 (**B**,**D**) was investigated in individuals with T1D (**A**,**B**) and T2D (**C**,**D**) recruited to the DARE (Diabetes Alliance for Research in England) study. C-peptide levels were compared for each genotype in both variants. (**E**). Minor allele frequencies (MAF) and Hardy–Weinberg equilibrium (HWE) for each variant. * *p* < 0.05 compared with corresponding control (Mann–Whitney test).

C-peptide was used as a marker of residual β cell function and the DARE study cohort was stratified by genotype. In participants with T1D, the rs10517086_A variant was associated with lower residual C-peptide when compared with other genotypes (Figure 8A; *p* < 0.05), but this effect was not observed for any rs1534422 genotype (Figure 8B). An effect was not observed for either variant in participants with T2D (Figure 8C,D).

## 4. Discussion

T1DM is characterised by β cell death and impaired insulin production, largely attributed to chronic inflammation and the presence of inflammatory infiltrate in the islets. The TEDDY study has identified two variants, rs10517086 (long intergenic non-protein coding RNA 2357 (*LINC02357*) and rs1534422 (*MIR3681* host gene (MIR3681HG)), as markers for an increased risk of developing T1DM, in addition to other autoimmune diseases such as multiple sclerosis [8,9]. It is predicted that both rs10517086 and rs15017086 have a role in the development of islet autoimmunity, even prior to T1DM development, with rs10517086 displaying an age-related effect on the risk of IA, with increased risk before age of 2 years [8,10]. It is not known how these variants confer their effect on the cells of the immune system and the β cells themselves. The present study found a direct role for rs1534422 and rs10517086 in negatively regulating the β cell secretory machinery, through increased inflammatory cytokine production and altered transcriptional regulation.

Since little was known about whether these variants directly impact the β cell or create an inflammatory environment that is harmful to the β cell, we introduced each variant into the BRIN-BD11 β cell line and added conditioned media from variant expressing Jurkat T cells to BRIN-BD11 cell cultures to model the inflammatory environment. In line with previous computational based studies, we reported that the presence of rs1534422 and rs10517086 negatively impacts β cell function and viability [8,10]. We have shown that the rs1534422 variant leads to an increase in β cell apoptosis. We also reported that both rs1534422 and rs10517086 reduced glucose stimulated insulin secretion and provoked changes in several β cell genes and transcription factors that are needed for normal function.

Most of the changes in function observed in this study occurred irrespective of whether the variants were expressed directly in β cells or whether conditioned media from a T cell line was applied to the β cells. This suggested that soluble secreted factors released from both cell types may drive β cell transcriptional regulation and function. We have previously shown that TNFAIP3 deficient β cells release higher concentrations of TNF-α, IL-1β and IFNγ, and that these cytokines independently drive reductions in many β cell markers and transcription factors [15]. In the current study, media obtained from rs1534422 and rs10517086 expressing BRIN-BD11 cells and Jurkat T cells had a significantly increased content of pro-inflammatory cytokines, including TNF-α, IL-1β, IL-6 and IFNγ, and a moderate decrease in anti-inflammatory cytokines, including IL-10. The release of pro-inflammatory cytokines could largely explain the mechanism behind the observed reduction in β cell function and increase in inflammation in the presence of these variants. However, HNF1A was significantly upregulated in the presence of either variant and our past data do not support a role for these cytokines in this observation [15].

HNF1A is needed for correct β cell function. However, overexpression of HNF1A compromises islet morphology, inhibits β cell cycle activity and induces cell death [17]. HNF1A is a master regulator of cellular processes. Mutations in *HNF1A* are associated with diabetes development [18,19], especially in monogenic forms of the disease [20]. Furthermore, *HNF1A* regulates expression of transcription factors including p53 binding proteins [21], which in turn have been shown to regulate glucose homeostasis [22,23]. In the current study, knockdown of *HNF1A* in wild-type β cells predictably led to a decrease in the expression of all investigated β cell markers and several transcription factors. *RelA* and *NFKB1* were not affected by *HNF1A* knockdown, which is consistent with data from our apoptosis assay. Of note, knockdown of HNF1A in rs1534422 and rs10517086 variant expressing BRIN-BD11 cells led to normalisation of previously affected genes and a reduction in apoptosis.

Ensemble modelling has determined that the variants lie in the gene desert and are intronic long non-coding RNAs (lncRNA). Normal cellular functions and transcriptional regulation are controlled to some extent by lncRNAs, but all of the mechanisms are not fully understood, and this does not take into consideration the role of the variants hosted by those lncRNAs [24]. Non-coding region variants can, however, serve as genetic and physical waypoints for comparative genomic investigations [25]. Although the mechanisms remain unclear, we have shown that induction of the variants significantly increased the expression of their associated lncRNAs, which was normalised upon *HNF1A* silencing.

**Figure 9:**
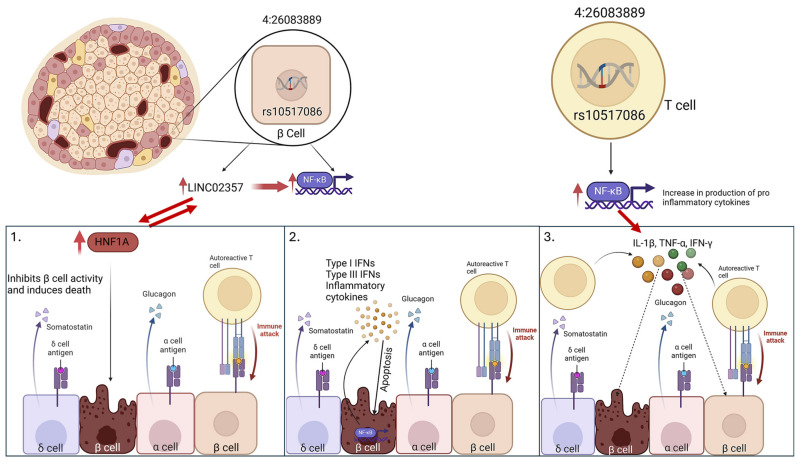
Mechanisms of rs15017086 action in the β cell. rs10517086 leads to a triple insult to the β cell by upregulating *HNF1A* expression through upregulated *LINC02357*, enhancing cytokine release from the β cell itself, and by creating an inflammatory environment by T cells.

These in vitro data suggest that rs1534422 and rs10517086 exert a multiple insults on the β cell through excessive upregulation of *HNF1A* and induction of pro-inflammatory cytokines (as summarised in Figure 9). This is further exacerbated by the remarkably similar impact that the variants have when expressed in the β cell directly and when conditioned media from T cell models expressing the variants is used. Should these observations translate to in vivo models, the direct impact on the β cell and the further creation of an inhospitable environment surrounding the β cell will likely explain much of the risk of T1DM development associated with these variants.

## Figures and Tables

**Table 1:** Custom designed gRNA and ssOligo donor sequences.

Variant ID	gRNA Sequence	ssOligo Donor (5′-3′) Sequence
rs1534422	AGTAACATCTGACGGTGTAT	‘TZZGTCATTAAGTTTGATCGCCTCTTCTTTCAGCACCTGAGACTGTCTCCTTTCCACCCATACACTGTCAGATGTTACTTGGCATTAATGGAGTTTTGFET’
rs10517086	TGGAAGGTTGTCATAAACTC	AEZCTCAGTCCCTGAAATATAACAATGAGACGGAACTCCTGTGTCCCTGAGTTTATGACAACTTTCCAAGACCATCCTTTTTGTAAAAAATATATATFZA

**Table 2:** qPCR custom designed probes.

Target Gene	Probe Assay ID	Manufacturer
*18S*	502300	Roche
*TNFAIP3*	Rn01766081_m1	Invitrogen (Waltham, MA, USA)
*ABCC8*	506195	Roche
*KCNJ11*	506200	Roche
*KCNQ1*	506506	Roche
*GCK*	Rn00561265_m1	Invitrogen
*SCL2A2*	506188	Roche
*HNF1A*	500240	Roche
*Pdx1*	506832	Roche
*Nkx6.1*	506820	Roche
*Ngn3*	Rn00572583_s1	Invitrogen
*NFKB1*	500911	Roche
*RELA*	500723	Roche

**Table 3:** Oligo primer sequences.

Primer	Sequence
Glyceraldehyde-3-phospahate dehydrogenase	5′ CATGTTCGTCATGGGTGTGAACCA 3′ 3′ ATGGCATGGACTGTGGTCATGCGT 5′
MIR3681HG	5′ CAGAGAGCATGGGTCAGCTT 3′ 3′ TCTCTTCAATGCCCCGTGAG 5′
LINC02357	5′ TTCCTGAAGCCTCCTGGTTTG 3′ 3′ CTCCATGGTTTCAGCTGTCC 5′

## Data Availability

All in vitro data are presented within the manuscript and available from the corresponding author upon reasonable request. The clinical datasets analysed in this study may be accessed through an application to the Peninsula Research Bank, which is managed by the NIHR Exeter Clinical Research Facility. Information on application or data are available on http://exeter.crf.nihr.ac.uk/content/tissue-banks (accessed on 20 October 2024).

## Supplementary Materials

The following supporting information can be downloaded at: https://www.mdpi.com/article/10.3390/genes16020172/s1, Figure S1: The insertion of CRISPR constructs into BRIN-BD11 and Jurkat T cells was confirmed via three independent methods; Figure S2: rs10517086 and rs1534422 were induced in BRIN-BD11 cells and the presence of the variants confirmed visually through the fluorescent GFP tag; Figure S3: BRIN-BD11 cells were treated with 30 ng siRNA against HNF1a or a negative control (scrambled siRNA) and the expression of HNF1a mRNA measured by qPCR.
