# Supplementary material for: Type 1 Diabetes Risk Variants Reduce Beta Cell Function
Source: Genes (Basel). 2025 Jan 29;16(2):172. doi: 10.3390/genes16020172 (PMC11855905; doi:10.3390/genes16020172)
Supplement: Supplementary file 1 [file genes-16-00172-s001.zip › genes-3376781-supplementary.pdf]

ELECTRONIC SUPPLEMENTARY MATERIAL

Type 1 Diabetes risk variants reduce beta cell function

Wiktoria Ratajczak,[1] Angus G. Jones,[2] Sarah D Atkinson,[1] Catriona Kelly[1*]

1. Personalised Medicine Centre, School of Biomedical Sciences, Ulster University, C-TRIC Building, Altnagelvin Hospital Campus, Glenshane Road, Derry/Londonderry, Northern Ireland, UK
2. University of Exeter Medical School, RILD Building, RD&E Hospital Wonford, Barrack Road, Exeter, UK

*** Corresponding author:** Dr Catriona Kelly, Personalised Medicine Centre, School of Medicine, Ulster University, C-TRIC Building, Altnagelvin Hospital Campus, Glenshane Road, Derry/Londonderry, Northern Ireland, UK. c.kelly@ulster.ac.uk

Short title: Functional effects of T1D SNPs

Key Words: Type 1 diabetes, risk variants, beta cells, inflammation

Fig. S1. Validation of CRISPR constructs insertion

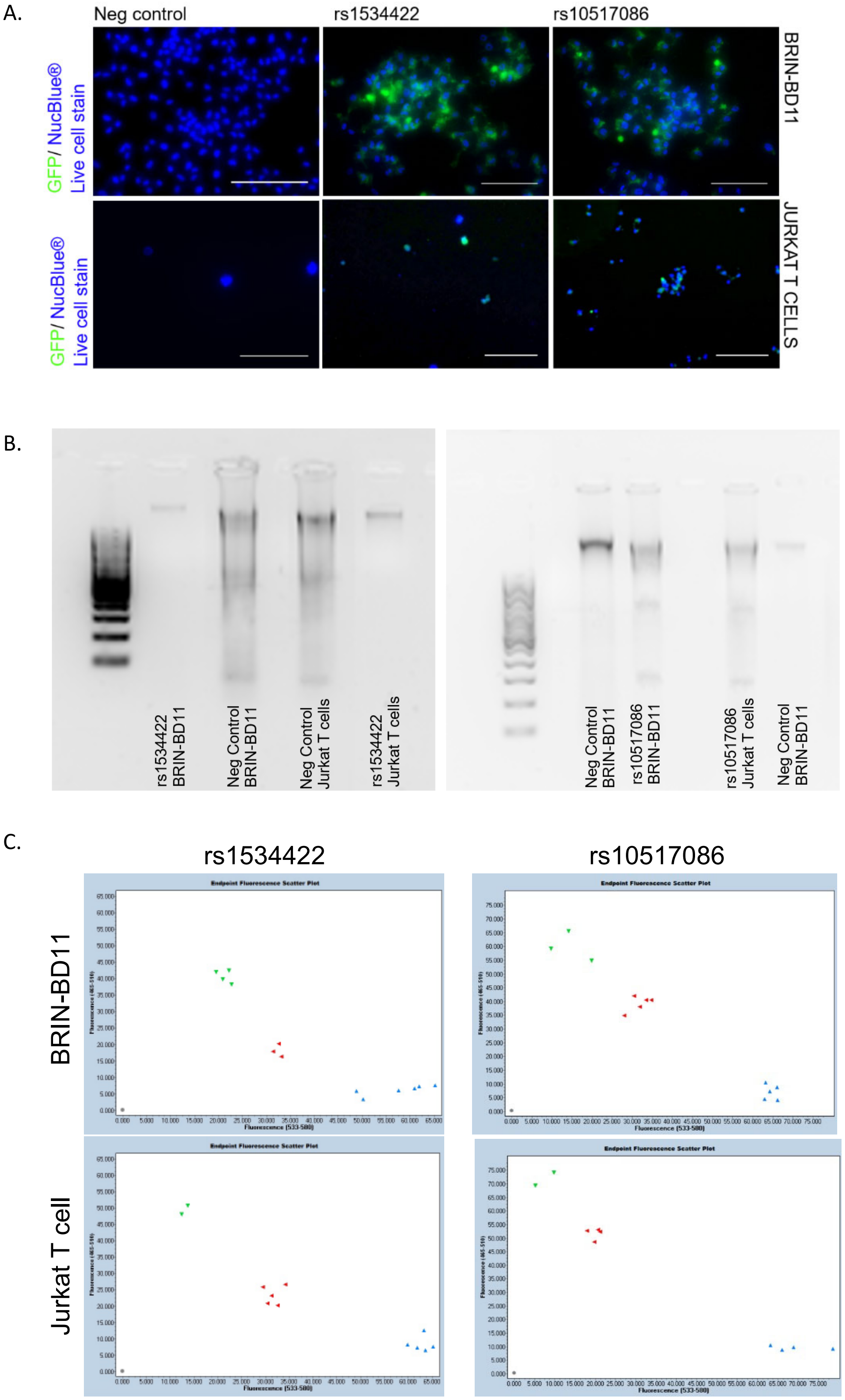

The insertion of CRISPR constructs into BRIN-BD11 and Jurkat T cells was confirmed via three independent methods. Visual inspection for the presence of the fluorescent GFP tag **(A).** Restriction digest enzymes TspRI (rs1534422), HPY188III (rs10517086) were used to establish presence or absence of wild type/variant type **(B).** Part of cell population for each cell type and treatment (variants and controls) was harvested and DNA extracted. The pool of DNA for each condition and cell type was screened through end point genotyping with independent TaqMan genotyping probes (rs1534422, assay ID: C____418159_10; rs10517086, assay ID: C__30562980_20). Cells expressing the variants were expanded for functional studies (Grey circle- water control; Green triangle -Test samples: Homozygous for allele 2; Red triangle – Test samples: Heterozygous for both alleles; Blue triangle – Known Negative controls – homozygous for allele 1) (**C**).

Fig. S2. Maintenance of variants upon further *HNF1a* silencing

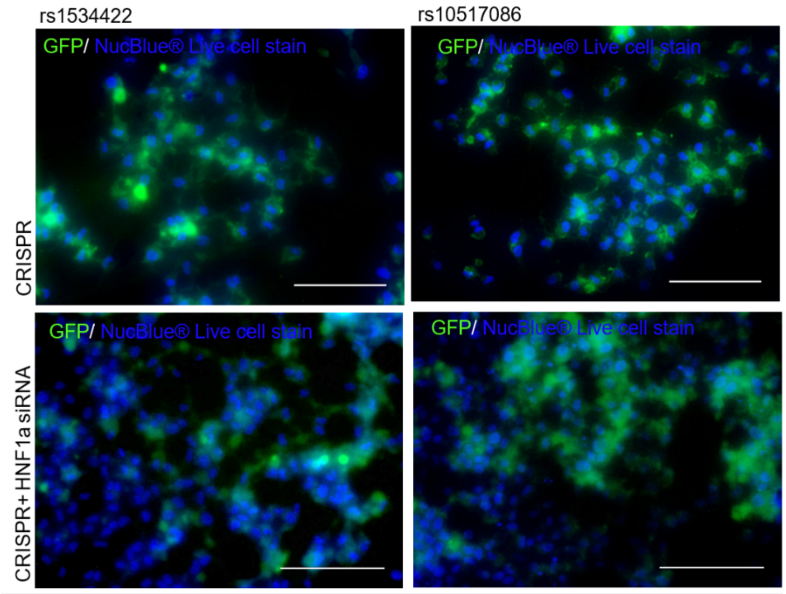

rs10517086 and rs1534422 were induced in BRIN-BD11 cells and the presence of the variants confirmed visually through the fluorescent GFP tag. The cells were allowed to recover for 48 hours and then were treated with 100 ng siRNA against *HNF1a* for 72 hours. Both variants were stably present in BRIN-BD11 cells following *HNF1A* silencing - confirmed by presence of GFP tag

Fig. S3. Transfection efficiency

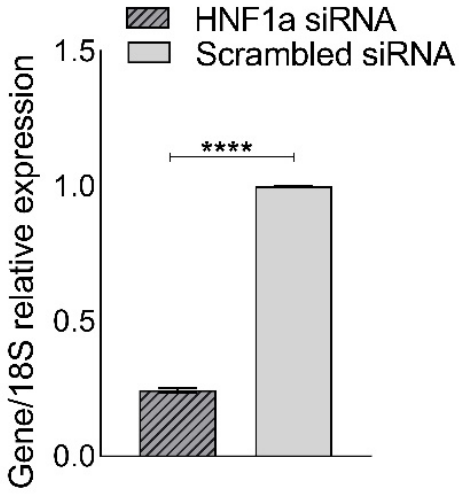

BRIN-BD11 cells were treated with 30 ng siRNA against *HNF1a* or a negative control (scrambled siRNA) and the expression of *HNF1*a mRNA measured by qPCR. Relative expression against 18S was calculated using 2^ΔCt. Data are presented as mean ± SEM (n = 4 independent experiments). ****P<0.0001 compared with negative control (*t*-test).
